# PD149 Comparison Of The Draft European Union Health Technology Assessment Template With Germany’s AMNOG Template

**DOI:** 10.1017/S0266462324003829

**Published:** 2025-01-07

**Authors:** Eva-Maria Reuter, Thomas Klein-Hessling, Carolin Rittmeyer, Maria Katharina Schweitzer, Sebastian Werner

## Abstract

**Introduction:**

European Union Health Technology Assessment (EU HTA) aims to use resources more efficiently, ensure high quality assessments, and promote the widespread availability of medicinal products. We compared the draft EU HTA template (EUnetHTA21 submission dossier template) with Germany’s Arzneimittelmarkt-Neuordnungsgesetz (AMNOG) template to assess their conformity and to estimate whether the draft EU HTA template requires more or less effort than the AMNOG template.

**Methods:**

Four experts (two statisticians and two medical writers) independently compared 39 categories of both templates following the four-eyes principle. The categories were defined based on the formal and methodological requirements as well as on the specific instructions regarding content, data presentation, and documentation in both templates. We assessed each category for conformity between templates (low, medium, high) and determined the EUnetHTA21 template’s additional effort (much less, less, equivalent, more, much more) relative to the AMNOG template. The comparison was carried out under the assumption that only a single research question on a population, intervention, comparator, and outcome (PICO) would be addressed in both submission procedures.

**Results:**

We found that the draft EUnetHTA21 template and the AMNOG template had substantial conformity in most categories (21/39), indicating comparable or identical formal and methodological requirements. For 10 of the 39 categories the conformity between templates was rated as medium. Low conformity was found for eight of the 39 categories, including categories outside one template’s scope. For most categories (20/39) we expect an equivalent effort per PICO. More or much more effort is expected for 13 of the 39 categories. For only six of the 39 categories, less or much less effort is expected for the draft EUnetHTA21 template, compared with the AMNOG template.

**Conclusions:**

The analysis highlights strong similarities between the templates but shows increased effort per PICO with the draft EUnetHTA21 template. This effort will be further increased with multiple PICO questions. This could challenge the feasibility of the EU HTA process, especially considering the short timeframe required. The template comparison will be updated once the final EU HTA template is available.

